# Synchronous papillary thyroid cancer and non-Hodgkin lymphoma: Case report

**DOI:** 10.1097/MD.0000000000009831

**Published:** 2018-02-09

**Authors:** Georgi I. Popivanov, Pavel Bochev, Radka Hristoskova, Ventsislav M. Mutafchiyski, Mihail Tabakov, Anthony Philipov, Roberto Cirocchi

**Affiliations:** a Clinic of Endoscopic, Endocrine surgery and Coloproctology; b Clinic of Nuclear medicine, Medical University, Varna; c Department of Pathology, Military Medical Academy; d Clinic of Abdominal surgery, University Hospital for Active treatment, Sofia, Bulgaria; e General and Surgical Oncology Division, University of Perugia, Perugia, Italy.

**Keywords:** non-Hodgkin lymphoma, papillary thyroid cancer, treatment

## Abstract

**Rationale:**

Differentiated thyroid cancer is the most common endocrine malignancy with concomitant hematological malignancy in 7%.

**Patient concerns:**

We present a case of a synchronous papillary thyroid cancer and a follicular variant of non-Hodgkin lymphoma and discuss the possible diagnostic and treatment dilemmas.

**Diagnosis:**

A 48-year-old female was reffered to our hospital with diagnosis „thyroid cancer“. Due to a history compatable of synchronous lymphoproliferative disease we performed a computed tomography, which revealed multiple enlarged lymph nodes in the neck, mediastinum, axilla and abdomen.

**Interventions:**

A total thyroidectomy with dissection of the central compartment was performed. The microscopic examination of thyroid gland revealed multifocal papilary thyroid cancer and metastaes from the same cancer plus aggressive follicular B-cell non-Hodgkin lymphoma in the lymph nodes. Despite the classic approach „solid cancer first“, due to the advanced stage of lymphoma we first started the chemotherapy of NHL. She received 8 cycles of CHOP and I^131^ therapy with 129 mCi. Because of incomplete response 4 cycles Mabthera plus Bendamustin were added. The follow-up PET scan revealed complete remission of lymphoma and bilaterally enlarged single cervical lymph nodes, previously known to be iodine positive on I^131^-SPECT/CT. She was sheduled for bilateral radical neck LND.

**Outcomes:**

Complete remission of NHL and residual single metastatic cervical lymph nodes requiring bilateral radical neck LND.

**Lessons:**

The synchronous DTC and NHL is rare. To date, there is no standardized approach due to lack of experience. We suggest lymphoma first approach with synchronized and tailored multidisciplinary efforts. The molecular mechanisms of this link are poorly understood and yet remain to be elucidated.

## 1 Introduction

Differentiated thyroid cancer (DTC) is the most common endocrine malignancy with a significant increased incidence during the last decades. In USA it has raised from 4.9/100,000 in 1975 to 14.3/100,000 in 2009.^[1]^ To a large extent this change resulted from an increased incidence of papillary thyroid cancer (PTC), which accounts for about 90% of all DTC. Probably it is due to improved diagnostics of the early cancer <1 cm.

We present a case of a synchronous PTC and a follicular variant of non-Hodgkin lymphoma (NHL) and discuss the possible diagnostic and treatment dilemmas.

## 2 Case presentation

**Figure 1:**
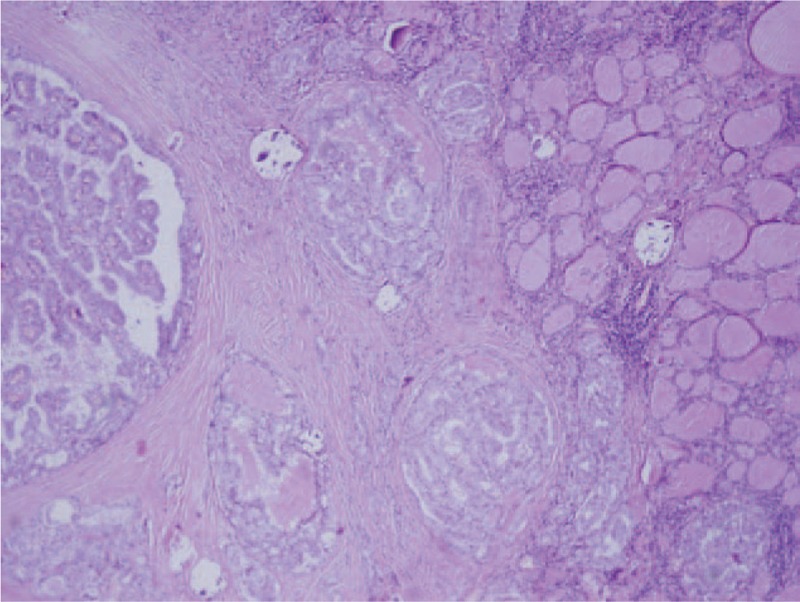
Microscopic view—papilary thyroid cancer with intact thyroid capsule (HE, × 4).

A 48-year-old woman was referred to our hospital with an initial diagnosis “thyroid cancer.” Due to rapidly growing neck lump she underwent ultrasound examination revealing thyroid nodule with diameter 12 mm and conglomerates of multiple enlarged cervical lymph nodes bilaterally. The prehospital fine needle biopsy raised suspicion of thyroid cancer due to high thyreoglobuline level. After admission, due to a history of fever up to 39 °C, marked weight loss, night sweats, and dyspnoe, a synchronous lymphoproliferative disease was suspected. So, we performed a whole body computed tomography (CT) which revealed multiple enlarged lymph nodes in the neck, mediastinum, axilla, and abdomen without organ involvement (Fig. 1). We started with a biopsy of left cervical lymph nodes at level V which proved concomitant metastasis from PTC and NHL. Trepanobiopsy showed normal bone marrow. The serum levels of thyreoglobuline and β2-microglobulin were 300 and 6.4 mg/L, respectively.

**Figure 2:**
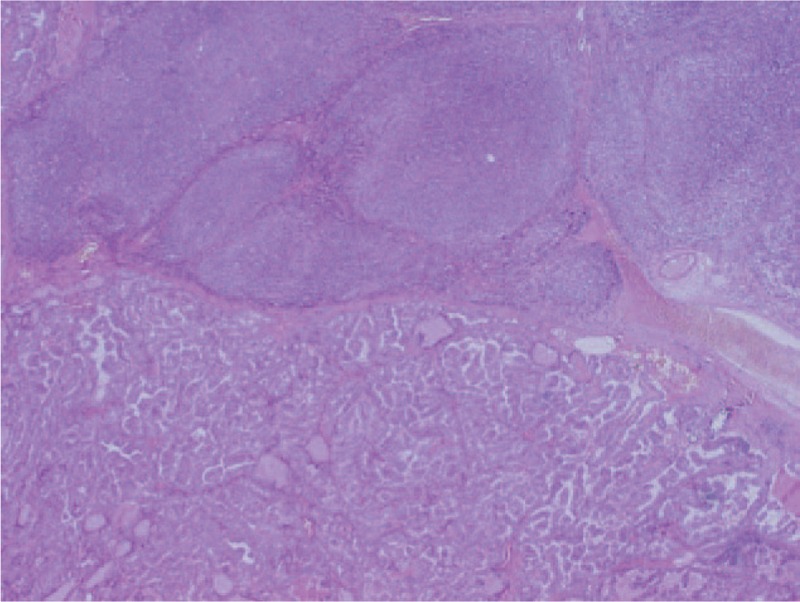
Compartment VI lymph node – synchronous metastasis from DTC and follicular B-cell NHL with transition to large B-cells lymphoma (HE, × 2). DTC = differentiated thyroid cancer.

After multidisciplinary discussion we performed a total thyroidectomy with dissection of the central compartment (level VI). The microscopic examination of thyroid gland revealed multifocal papillary thyroid cancer with diameter of the largest nodule 15 mm, multiple ones with size 2 to 3 mm and intact thyroid capsule. The paratracheal lymph nodes were engaged by metastases from the same cancer plus aggressive follicular B-cell NHL with transition to large B-cells lymphoma, grade 3b (follicular lymphoma prognostic index—2) (Fig. 2). The subsequent scintigraphy with I^131^ and single-photon emission computerized scan (SPECT)/computed tomography (CT) revealed multiple unilateral metastases in the cervical lymph nodes within the levels III, IV, and V, and limited contralateral metastases in levels IV and V without a distant spread in lung and bones. PTC was considered as T1bN1bM0, stage IVa due to the age of the patient, while NHL was staged as III B.

**Figure 3:**
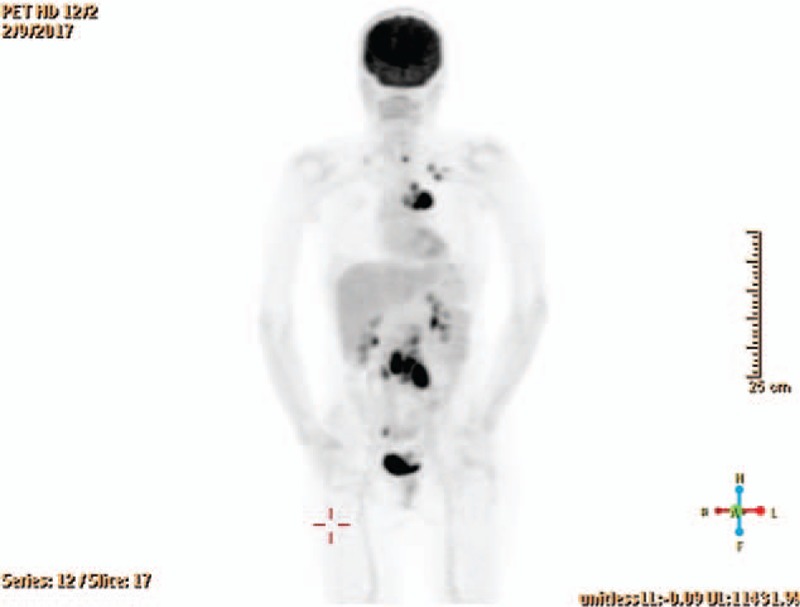
Index FDG-PET/CT scan positive for nodal involvement above and below diaphragm, positive cervical lymph nodes restricted to left side.

**Figure 4:**
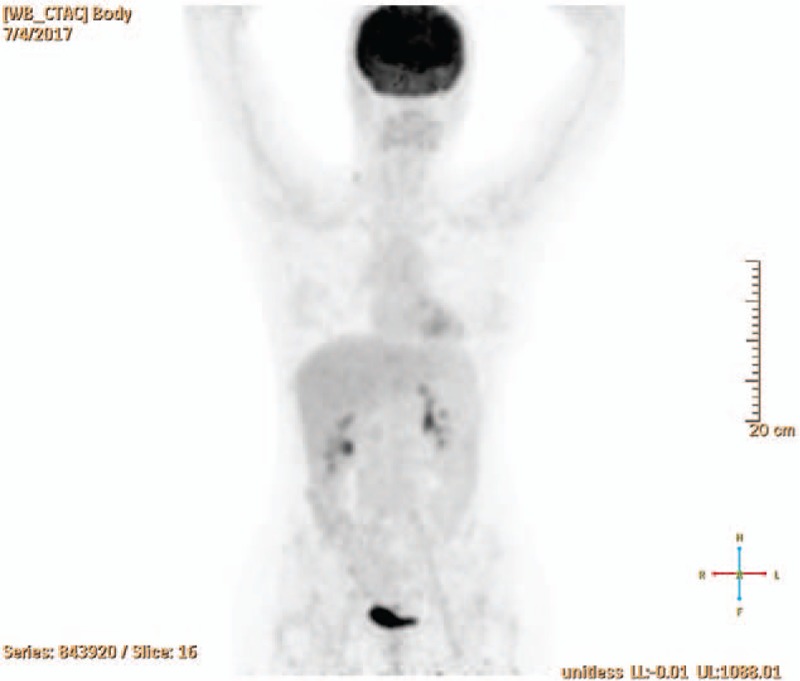
Follow-up FDG-PET/CT scan after 8 cycles CHOP and I^131^ therapy with 129 mCi consistent with complete remission of the NHL (according to Lugano response criteria). CHOP = cyclophosphamide, hydroxydaunorubicin, oncovin, prednisolone, FGD-PET = fluorodeoxyglucose-positron emission tomography.

**Figure 5:**
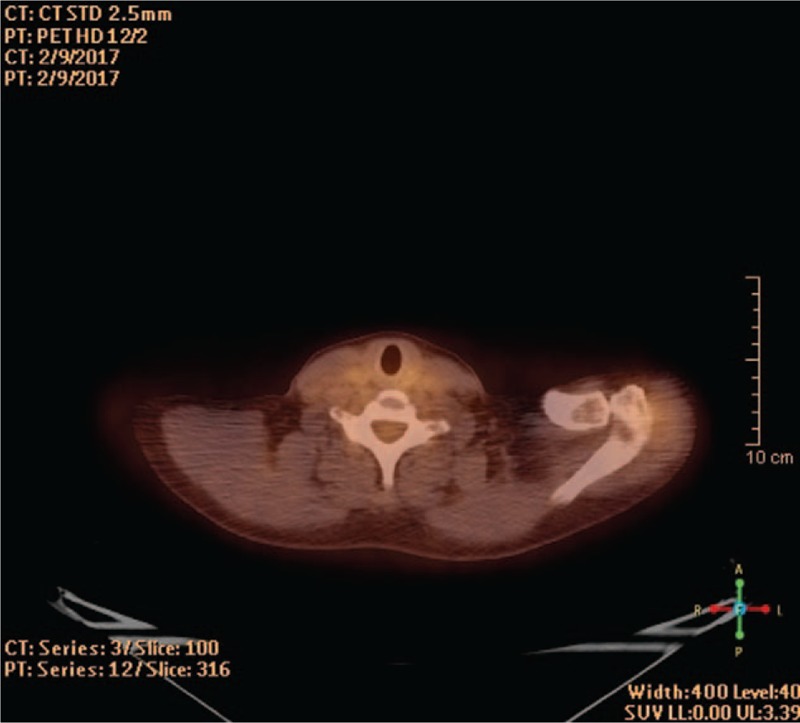
Follow-up FDG-PET/CT scan—enlarged, but PET negative lymph nodes in the right cervical region (known to be iodine positive on post-treatment SPECT CT) consistent with DTC lymph node metastases.

The patient received 8 cycles of CHOP and I^131^ therapy with an overall dose 129 mCi. Because of incomplete response with persistance of enlarged lymph nodes in supraclavicular area, mediastinum, and retroperitoneum on the fluorodeoxyglucose-positron emission tomography (FGD-PET) scan (Fig. 3) she proceeded with 4 cycles Mabthera plus Bendamustin. The follow-up PET scan revealed complete remission of NHL according to Lugano response criteria (Fig. 4) and bilaterally enlarged cervical lymph nodes, previously known to be metastatic/iodine positive on ^131^I-SPECT/CT (Fig. 5). A 2-year maintenance therapy with Mabthera was set (4 cycles per year) and the patient was scheduled for bilateral radical neck LND.

The Institutional Ethics Committee approved the publication of the case (prof. Julian Rainov, prof. Ventsislav Mutafchiyski, prof. Rumen Popov, Assoc. Prof. A. Donchev, d-r Vesel Kantarjiev, Albena Chamurjieva).

## 3 Discussion

According to a large Italian cross-sectional study comprising 6386 females, thyroid disease is associated with an increased risk for extra-thyroid malignancy with odds ratio (OR) 3.2. The incidence of the concomitant hematological malignancy was 7%. DTC was associated with significantly higher risk (OR 12.2), most pronounced in the age 0 to 44 (OR 23.8).^[2]^ We found only 2 reports in the English literature reporting simultaneous DTC and NHL. Rizkallah et al^[3]^ reported similar case, whereas Singh et al^[4]^ published recently a case with triple malignancies—laringeal cancer, DTC, and NHL. Although it is different from our case, Guzzo et al^[5]^ reported 33 cases with synchronous DTC and squamous cell carcinoma of head and neck for the period 1975 to 2004, of which 31 were diagnosed during the pathological examination.

The present case clearly demonstrates the challenges regarding the sequence of the treatment procedures, the time interval between them and the time and extent of cervical lymph node dissection (LND). According to the literature the prophylactic central compartment LND is not warranted and should be reserved only for the cases with macroscopic lymph node metastases as in our case.^[1,6–8]^ The recent guidelines^[1,6]^ recommend compartment oriented LND in such cases. Uchino et al^[9]^ advocated modified dissection of the central and ipsilateral lateral compartments. However, in cases as ours the dilemma is how to know whether LN are metastatic or are primarily affected by NHL. The I^131^ scintigraphy was performed after thyroidectomy to avoid radioactivity interference with the primary tumor, thus allowing a better visualization of the possible metastases from PTC and facilitating the postoperative I^131^ treatment. Despite the histologically proven contralateral metastases in level V in our case, we were afraid of performing of lateral dissection due to the worsened general condition and the possible delay of the NHL therapy in case of major complication. On other hand, certain authors advise seeking of “a balance between oncological benefit and surgical risk” due to the common dysfunction of the lateral neck nerves.^[10]^ Moreover, it has been demonstrated that secondary LND is a safe procedure.^[11]^

Despite the classic approach “solid cancer first,” due to the relatively good prognosis of PTC^[4]^ and the advanced stage of NHL with a pronounced clinical manifestation and aggressive course, we first started the chemotherapy of NHL similar to other authors.^[3]^

Several studies, as cited by Prinzi et al,^[2]^ attempted to explain the link between DTC and extra-thyroid malignancy implicating that “the long-term carcinogenic effects of specific cancer treatments might be responsible for a second cancer.” However, in our case there were synchronous occurrence of 2 malignancies and the most plausible explanation is the presence of yet unidentified molecular link or presence of general vulnerability carrying a higher risk for malignant transformation.

## 4 Conclusion

The synchronous DTC and NHL is a rare condition, which may pose significant diagnostic and treatment dilemmas. To date, there is no standardized approach due to lack of experience. We suggest “lymphoma first approach” with synchronized and tailored multidisciplinary efforts. The molecular mechanisms of this link are poorly understood and yet remain to be elucidated.
